# Directed Donation: Special Considerations and Review for Contemporary Clinical Practices

**DOI:** 10.31486/toj.20.0068

**Published:** 2021

**Authors:** Gordon Wadge, Jenny Zhang, John Seal, Edgar Shannon Cooper, Caroline R. Alquist

**Affiliations:** ^1^ Department of Hematology and Oncology, Ochsner Clinic Foundation, New Orleans, LA; ^2^ Department of Pathology, University of Arizona, Banner – University Medical Center Tucson, Tucson, AZ; ^3^ The University of Queensland Faculty of Medicine, Ochsner Clinical School, New Orleans, LA; ^4^ Section of Abdominal Transplant, Department of Transplant Surgery, Ochsner Clinic Foundation, New Orleans, LA; ^5^ Section of Transfusion Medicine and Histocompatibility, Department of Pathology and Laboratory Medicine, Ochsner Clinic Foundation, New Orleans, LA; ^6^ Hoxworth Blood Center Academic Unit and Department of Pathology, University of Cincinnati, Cincinnati, OH

**Keywords:** *Blood component transfusion*, *blood donors*, *blood transfusion*, *directed donation*

## Abstract

**Background:** Directed blood donation is defined as the donation of blood or its components for the purpose of transfusion into a specified individual. Directed blood donation holds historic significance, and although practices as of 2021 encourage voluntary, nonrenumerated blood donations, public interest in directed donation remains. Requests to discuss the risks and benefits of directed donations are a common inquiry for transfusion medicine, transplant, and hematology/oncology professionals. This narrative review discusses the history of directed donation and summarizes directed donation considerations in the context of modern transfusion practices.

**Methods:** We conducted a systematic search of PubMed for published literature on the topic of directed blood donation and gathered information about its benefits and potential harms with respect to the variety of products used in transfusion medicine.

**Results:** The drawbacks of directed donation include transfusion-transmitted infection risk, alloimmunization risk, increased transfusion-associated graft vs host disease risk, decreased expediency in treatment, and increased administrative burdens. However, a role remains for directed blood donation in specific patient populations, such as individuals with rare blood types or immunoglobulin A deficiencies, because of the difficulties in finding compatible blood for transfusion.

**Conclusion:** Clinicians should consider the risks and benefits when discussing directed blood donations with patients and family members.

## INTRODUCTION

Directed blood donation is defined as the donation of blood or blood components for the purpose of transfusion into a specified individual, generally a recipient identified in advance of the collection. Directed blood donation has historic precedent. The first documented successful blood transfusion in 1818 was a directed donation from a husband to his postpartum wife.^1^ While this directed donation was born of necessity, transfusion practices as of 2021 encourage voluntary, nonrenumerated blood donations.^2^ However, public interest in directed donations remains, and requests to discuss the risks and benefits of directed donations are a common inquiry for transfusion medicine, transplant, and hematology/oncology professionals. This narrative review discusses the history of directed donation and summarizes directed donation considerations in the context of modern transfusion practices.

Autologous donation is outside the scope of this review, but discussions of autologous donations should include blood conservation techniques, intraoperative cell saver technology, historic transfusion requirements by procedure type, and the typical need for >1 unit when transfusion is required.^3^ Autologous donations are routinely used in low-risk procedures, such as bone marrow donations, that historically require single-unit postoperative transfusions.

## METHODS

We conducted a systematic search of PubMed for published literature on the topic of directed donations and also examined the reference lists of the articles. Topics searched included the history of transfusions, altruism, directed donations, hemophilia and other coagulopathies, and alloimmunization. We gathered information about the history of directed donations and the benefits and potential harms of directed donation with respect to the variety of products used in transfusion medicine.

## PSYCHOLOGY OF BLOOD DONATION

In 1970, British social scientist Richard Titmuss published *The Gift Relationship: From Human Blood to Social Policy*. This book greatly influenced both British and US transfusion practices by asserting that blood donation must occur as an altruistic act to avoid human exploitation, commercialism, and risk.^4,5^ Titmuss warned that paid donor arrangements could result in wasteful and inefficient donation systems with exploitative redistribution of blood products. He predicted that a nonaltruistic path would contribute to the degradation of society via enhanced selfish interests and personal gain.^4,5^

With respect to blood donation, 2 forms of altruism must be considered: induced altruism and kin altruism. Induced altruism is defined as giving without expected reciprocal benefit.^4,5^ Voluntary, nonrenumerated blood donations are an example of induced altruism. Donated products enter the anonymized pool of available blood without reference to their eventual recipient. Kin altruism is defined as increased altruistic action if the person aided is known to the giver.^4,5^ Kin altruism is uniquely satisfied by directed donation and is a driving influence on directed donation behavior. In general, studies identify altruism as the primary reason for modern blood donations.^6^ Other motivating factors behind the decision to donate include gratitude, reciprocity, replacement, duty, having a rare blood group, benefit (eg, discovery of blood type), and personal appeal.^4,5^

Directed donations may be influenced by extrinsic pressures, such as coercion or monetary compensation, particularly in times of crisis.^2^ Ramifications of such pressure may include inaccurate answers on predonation screening questions. The US Food and Drug Administration (FDA)–approved Uniform Donor History Questionnaire (UDHQ) standardizes blood donor screenings.^6^ Questions about high-risk behaviors of the donor include male-to-male sex and intravenous drug use. Reports have shown, however, that donors have sometimes answered untruthfully because of donor perception of their own blood safety, their faith in the fidelity of infectious disease testing, confidentiality concerns, and their altruistic desire to save lives.^7,8^ UDHQ high-risk behaviors are correlated with transfusion-transmissible diseases such as hepatitis and HIV. In the case of a directed donation, donors may be pressured to answer the screening questions untruthfully because they are being relied upon by the intended recipients.

Additionally, infectious disease testing is not foolproof. Testing modalities combine serology testing with nucleic acid technology testing, but infectious disease testing is not comprehensive, particularly for viruses with controversial transfusion-transmission risk, such as Epstein-Barr virus, and for emerging viral infections.^9^ False negatives can also occur, particularly during acute infection periods when host-response serologic markers have not yet developed.^9,10^ Thus, while the risk of transmission per unit transfused for viruses such as HIV and hepatitis B virus (HBV) is extremely low (1 in 1 million or lower for each), the risk is not zero, and further testing has been posited as ineffectual in maximally lowering the risk.^9,10^ The enhanced infectious disease risk of directed donation is further discussed in a subsequent section. While incorrect answers on the UDHQ are a potential issue for all donations, directed donations motivated by kin altruism may increase the risk of a donor answering the UDHQ untruthfully.

## HISTORIC NEED FOR DIRECTED DONATION

Historically, directed donation served to minimize donor exposure in frequently transfused patients. Patients receiving the bulk of frequent plasma and plasma-derivative transfusions include those with hemophilia and von Willebrand disease. Prior to treatments such as recombinant replacement factors and desmopressin, whole blood or plasma were the only available treatments for a bleeding episode, the frequency of which varied based on disease severity.^10^

Hereditary hemophilia is a set of X-linked disorders characterized by a lack of factor 8 in hemophilia A or factor 9 in hemophilia B. Frequent transfusions of plasma-derived products such as cryoprecipitate and plasma factor concentrates became synonymous with inadvertent transmission of viral-borne illnesses to the hemophilia population, including HIV, HBV, and hepatitis C (HCV). Among US hemophiliacs treated between 1982 and 1984, 74% of patients who received factor 8 concentrates and up to 90% who received factor 9 concentrates were positive for antibodies to HIV.^11^ Cirrhosis, likely secondary to hepatitis transmission, was also seen in 15% to 38% of patients with hemophilia.^12^ These infection rates may be attributed to the amplified risk of multiple donor exposures, paid plasma donation sources using nonaltruistic donors, and the lack of screening for HIV risk factors at that time.^13^ Effective recombinant replacement factors were not developed until the late 1980s, and patients with hemophilia no longer had to receive as many plasma and plasma-derivative transfusions, decreasing their exposure to transfusion-transmitted infections.^12^

Von Willebrand disease is secondary to a qualitative or quantitative defect in the von Willebrand protein, which is vital to platelet plug formation and prevention of the inactivation and clearance of factor 8. One of the first treatments for von Willebrand disease was cryoprecipitate derived from human plasma in 1964.^14^ As for patients with hemophilia, patients with von Willebrand disease also had a risk of acquiring viral-borne illnesses such as HIV, HBV, and HCV, although they did not require transfusions as frequently.^15^ Desmopressin was not found to be clinically useful in patients with type 1 von Willebrand disease, the most prevalent form, until 1977.^14,16^ As a result, treatment no longer relied on frequent cryoprecipitate transfusions, decreasing transfusion-transmitted infection risks. In 1981, Humate-P, a pasteurized formulation of factor 8 and von Willebrand factor, was developed to treat types 2 and 3 von Willebrand disease.^17^

Modern recombinant products and improved processing technology have greatly decreased infectious disease risk for patients with hemophilia and von Willebrand disease.

## TRANSFUSION-RELATED INFECTIOUS DISEASE RISK

Interest in directed donation, particularly from a friend or an acquaintance, is often linked to a misperception of increased safety. Directed donation inquiries reached an all-time high in the 1980s around the discovery of HIV and its blood-borne transmission.^18^ Requests for directed donations have since decreased as laboratory evaluation and transfusion have become safer.^18^ The FDA mandates that all US blood products intended for transfusion be tested for HBV, HCV, HIV-1, human T-lymphotropic virus (HTLV) types 1 and 2, *Trypanosoma cruzi* parasites, and Zika virus. The rate of contracting HIV from a transfusion is currently estimated to be 1 in 1.5 million, while transmission rate estimates for HBV and HCV are 1 in 1 million for HBV and 1 in 1.2 million for HCV.^19,20^

Despite improvements in infectious disease screening and testing, however, directed donations are associated with an increased risk of transfusion-transmitted infection compared to blood from anonymous volunteer donors.^18^ One retrospective study found reactive rates per 100,000 donations for volunteer vs directed donors as follows: HIV (2.9 vs 7.2), HBV (12.4 vs 40.1), HCV (32.2 vs 93), and HTLV (2.5 vs 18.6). The crude odds ratios for HIV, HBV, and HTLV transmission from directed donor blood products are significantly higher than those seen with volunteer donors.^18^ The increased risk of a transfusion-transmitted infection in directed donors remains even when the donor is a parent of the patient. Combined rates of HIV, HBV, HCV, HTLV, and syphilis reactivity in parental directed donations were 6.33% vs 1.3% for community donations.^21^ Thus, increased risk of transfusion-transmitted infections in directed donor blood products should be considered because the detection of such infections would preclude the donated blood products from use.

## ALLOIMMUNIZATION RISKS

All transfusions carry an alloimmunization risk to human leukocyte antigens (HLAs) that may impact future transfusion and transplant outcomes. The immune system uses cell-surface HLAs and antibodies to identify and destroy non-self cells. Exposure to non-self HLAs on transfusion-transmitted white blood cells may result in a host response of alloantibody formation to those non-self HLAs. More than 30% of patients requiring multiple transfusions will develop alloantibodies, although this rate may be lower in patients with compromised immune systems.^22^ The risk of developing alloimmunization after red blood cell transfusion is estimated to be 0.5% per unit transfused or 1.15% per transfusion episode.^22^ A risk of alloimmunization secondary to residual leukocytes exists with plasma transfusions. However, the process of removing leukocytes through filtration from a blood product before transfusion, known as leukoreduction, has been shown to mitigate alloimmunization risk.^23^ Alloimmunization may also occur secondary to platelet transfusions. In one study, alloimmunization occurred in 45% of non-leukocyte-reduced platelet transfusions.^24^ Even following leukoreduction, 18% of platelet transfusion recipients may become alloimmunized.^24^

Anti-HLA antibody formation to transfused blood products has implications for a patient's future response to transfusions and for potential solid organ and bone marrow transplant recipients. Patients who develop anti-HLA antibodies may no longer appropriately respond to platelet transfusions that carry the relevant HLAs on their cell surface. Additionally, the presence of anti-HLA antibodies may rule out potential bone marrow and solid organ donors. Alloimmunization can have deleterious effects on solid organ and bone marrow transplants, including early death, impaired engraftment/organ tolerance, and increased rejection.^25-27^

The risk of alloimmunization becomes particularly important to consider for family members asking to directly donate blood products to a patient. Family members are the most common donor sources for bone marrow and solid organ donation given the likelihood of patient blood type and HLA matching. The development of anti-HLA antibodies following a transfusion from a family member's directed donation may eliminate that family member and relatives with similar typing as donor sources for future bone marrow or organ transplants.

## TRANSFUSION-ASSOCIATED GRAFT VS HOST DISEASE

Transfusion-associated graft vs host disease (TA-GVHD) is the failure of a recipient's immune system to recognize donor lymphocytes in a transfused product as foreign, allowing donor cells to mount a destructive response against the recipient's lymphoid tissue. Typically manifesting within 30 days after transfusion, TA-GVHD symptoms include fever, nausea, vomiting, mucositis, diarrhea, gastrointestinal bleeding, skin lesions, hepatic dysfunction, and pancytopenia. Without any known effective treatments beyond supportive care, mortality from TA-GVHD is >90%.^28^

TA-GVHD most commonly occurs following blood donation from a related donor with an HLA profile similar to the recipient's and in populations with limited HLA diversity.^28^ Leukoreduction alone is not sufficient to prevent TA-GVHD. To prevent TA-GVHD, blood products from relatives and those with predictably similar HLA profiles undergo gamma irradiation. This treatment effectively damages lymphocyte DNA to prevent posttransfusion engraftment and proliferation capabilities.^28^ In Japan, where the first case of TA-GVHD was reported in 1955, TA-GVHD incidence dropped from 0.15% between 1981 and 1986 to 0 cases in 2000 and 2001 following implementation of measures such as irradiation.^28,29^

Most directed donations are subject to irradiation regardless of recipient relation, but TA-GVHD remains a theoretical concern with any cellular product transfusion. In some areas, access to an irradiator may not be available, which either precludes the use of blood products from those with similar HLA profiles or, if the blood products are used, increases the risk of TA-GVHD occurring. Irradiated units also raise concerns for elevated plasma potassium levels because of the hemolysis of red blood cells.^30^ Hyperkalemia poses a potential risk for certain populations such as neonates and patients with renal failure and should be taken into consideration.

## TESTING RESTRAINTS AND ADMINISTRATION DIFFICULTIES

As stated previously, the FDA mandates that all US blood products be tested for HBV, HCV, HIV-1, HTLV types 1 and 2, *Trypanosoma cruzi* parasites, and Zika virus.^19,20^ The turnaround time for this required testing is often overlooked when planning for directed donations. Directed donations are not immediately available for transfusion; their release is contingent upon testing completion and acceptable test results. Depending on the testing and product preparation process of the particular blood bank, the period from collection to product availability for transfusion may be >48 hours. Because of the turnaround time, directed donation is a less feasible option than the use of voluntary, nonrenumerated blood donations when a transfusion is needed expediently because the latter products would have already been tested and prepared for transfusion.

In addition, directed donations have increased administrative requirements. Facility-specific circumstances may require that blood products be donated and collected at a separate facility than where the patient receives the transfusion. In comparison, a facility would already have blood products from voluntary, nonrenumerated blood donations in stock. Extra care must be taken to ensure the directed donation units are appropriately labeled, tested, and cross-matched for the intended patient, transported to the patient's specific location, and transfused to the correct patient.^30,31^ Directed donations therefore involve administrative burdens as well as time constraints.

## MODERN APPLICATIONS OF DIRECTED DONATION

Directed donation continues to play an important, albeit limited, role in transfusion medicine. Individuals requiring HLA-matched platelets, granulocytes, and products for immunoglobulin A (IgA) deficiency and individuals with fetal and neonatal alloimmune thrombocytopenia or rare blood types all rely on directed donors.^31^

Patients with rare blood types and significant blood antigen alloimmunization rely on donors of compatible blood. In 1998, the American Rare Donor Program (ARDP) was developed and tasked with keeping a registry of rare blood donor information. Managed by the American Red Cross National Reference Laboratory for Blood Group Serology in conjunction with the AABB (formerly the American Association of Blood Banks), the function of the ARDP is to facilitate donor identification for patients with rare blood types and individuals with antibodies to high-frequency antigens or with multiple common antibodies.^32^ Rare blood types are those that match <1 in 1,000 donations.^32^ Very rare products are those identified in <1 in 10,000 donated units. Examples of such phenotypes include the Bombay (found in 1 in 2,000 to 1 in 13,000 donations) and Indian (found in 1 in 250,000 donations) phenotypes.^33^ Donor information is updated semiannually in the ARDP. When a rare or very rare blood type is requested, the ARDP requests testing of a patient's sibling(s) or initiates recruitment of registered rare donors for directed donation.^32^

The ARDP services for donor identification are also commonly used by patients with sickle cell disease who have a high propensity for developing alloantibodies. From January 2005 to June 2006, 33% of requests for red blood cell products were for alloimmunized patients with sickle cell disease.^34^ High alloimmunization rates (7% to 47%) in the United States have in part been attributed to the fact that the majority of blood donors in the United States are of European descent.^35^ Europeans have different allele frequencies than most patients with sickle cell disease. For example, C and E of the Rhesus (Rh) antigen group, K of the Kell (KEL) antigen group, Fy(a) of the Duffy (Fy) antigen group, Jk(b) of the Kidd (Jk) antigen group, and S of the MNS antigen group are more frequently encountered in persons of European decent than in persons of African descent.^35^ The ARDP plays a crucial role in matching patients with rare blood types or significant alloimmunizations with specific donors for directed donation.

Alloimmunization is a significant cause of platelet refractoriness. Patients are defined as platelet refractory when their platelet counts fail to respond appropriately to 2 separate platelet transfusions. Platelet refractoriness has immune and nonimmune causes. Immune causes include human platelet antigen (HPA), or more commonly, HLA antibodies. As previously mentioned, leukoreduction may decrease HLA alloimmunization risk, but the risk varies with underlying diagnosis, history of pregnancy, and transfusion exposures.^33,36^ Nonimmune causes of platelet refractoriness include sepsis, splenomegaly, disseminated intravascular coagulation, and medications.^37^

Once nonimmune causes are excluded, a platelet antibody screen should be performed to evaluate platelet-refractory patients. If the screen indicates an alloimmune cause, HLA-appropriate products can be identified via cross-matching of available ABO-identical platelet inventory or by seeking an HLA-compatible donor. To identify such donors, HLA typing of the recipient and potential donors and identification of preexisting recipient HLA antibodies must be performed. An appropriate platelet donor should have HLA-A and HLA-B types in common with the recipient, or at a minimum, lack the HLAs to which the recipient has produced antibodies.^38^ HLA-matched platelets can be difficult and expensive to obtain.^38^ HLA-typed donor databases are therefore crucial to the identification of altruistic donors who are willing to provide compatible products to a patient in times of need. Thus, for patients with rare blood types or alloimmunization, a directed donation would serve them better than the voluntary, nonrenumerated blood donations typically available at a blood bank.

A form of directed donation in current use is granulocyte transfusion. While their value and efficacy are controversial, granulocyte transfusions may be given prophylactically to patients with chronic granulomatous disease or with severe neutropenia from a prolonged infection that has failed multiple antibiotics.^39^ Transfusions provide patients time to mount their own responses to the infection.^40^ While most centers use family members as donors, volunteer donors may also come from the community to provide ABO-compatible granulocyte products.^41^ Prior to collection, donors are administered mobilization medications such as steroids and/or granulocyte colony-stimulating factor to increase circulating neutrophils in the peripheral blood.^41^ Directed donation of granulocyte products is not without risk to both donor and recipient. Donor mobilization medications have potential risks of thrombosis, splenic rupture, and anaphylactoid reactions. Risks to the recipient are a 10% rate of alloimmunization and a host of potential postinfusion reactions, including fever, chills, rash, shortness of breath, pulmonary distress, and cytomegalovirus infections.^42,43^ Once granulocyte transfusions are initiated, irradiated granulocyte infusions are usually given daily and require recruitment of multiple individual donors. This treatment modality hinges on the availability of willing and available donors because of the short, 24-hour storage life of granulocytes, making a directed donation more feasible than voluntary, nonrenumerated blood donation.

IgA-deficient patients may require directed donations because they can develop anti-IgA antibodies that result in an anaphylactic reaction to all plasma-containing blood products. As such, IgA-deficient patients require washing of cellular blood products, which replaces the blood product plasma with normal saline and in the process removes microaggregates, leukocytes, and plasma proteins, including IgA. If a patient is receiving plasma derivatives, the products must come from an IgA-deficient donor. IgA-deficient products can be ordered from blood product distributors who can contact willing prescreened donors.

Fetal and neonatal alloimmune thrombocytopenia is a relatively rare condition that occurs when a mother develops antibodies against the paternal (non-self) HPAs on fetal platelets.^44^ Outcomes of this condition may include fetal intracranial hemorrhage and bleeding diatheses. Initial treatments of this condition—first performed in 1983—required directed donation of irradiated and washed platelets from the mother to the developing fetus through intrauterine transfusions.^44-46^ Current treatment of fetal alloimmune thrombocytopenia involves maternally administered intravenous immunoglobulin with or without steroids.^44^ Intrauterine platelet transfusions, maternally derived or special ordered, are now reserved as a second-line treatment. After birth, the primary treatment for neonatal alloimmune thrombocytopenia is platelet transfusion. The best platelets for transfusion are those from HPA-1bb/5aa-typed platelet donors who are contacted for donation. Newborns may also receive readily available random platelets or irradiated and washed maternal platelets with similar outcomes.^44^

## CONCLUSION

Inquiries about directed blood donation are common in clinical practice and present unique challenges. While induced altruism is the most common motivating factor for blood donations, the kin altruism behind directed donations introduces potential confounding motivations of gratitude, reciprocity, duty, and personal gain that can ultimately compromise the safety of the donation process. Modern practice favors the collection of blood products from voluntary nonremunerated donations because of the risks present in blood donations given for reasons other than altruism. However, improvements in blood product screening reduce that risk considerably, and specific risks associated with directed donation should be carefully considered. Providers and patients should be advised of directed donation drawbacks, including transfusion-transmitted infection, alloimmunization risk, increased TA-GVHD risk, decreased expediency in treatment, and increased administrative burdens.

While many requests for directed donations are rooted in historic concerns, a role remains for directed donations in practice. The best or only option for patients with rare blood types, IgA deficiency, or significant alloimmunization may be a directed donor.

## Notes

*This article meets the Accreditation Council for Graduate Medical Education and the American Board of Medical Specialties Maintenance of Certification competencies for Patient Care and Medical Knowledge.*
